# Development and effectiveness of a mobile phone application conducting health behavioral intervention among men who have sex with men, a randomized controlled trial: study protocol

**DOI:** 10.1186/s12889-017-4235-6

**Published:** 2017-04-24

**Authors:** Jin Yan, Aidi Zhang, Liang Zhou, Zhulin Huang, Pan Zhang, Guoli Yang

**Affiliations:** 1 The Third Xiangya Hospital, Central South University, Tongzipo Road 138#, Changsha, 40013 China; 2 Guangzhou Brain Hospital, Mingxin Road 32#, Guangzhou, 510370 China; 3 Changsha Center for Disease Control and Prevention, Weier Road 149#, Changsha, 410001 China

**Keywords:** Men who have sex with men, Mobile phone application, Behavioral intervention, HIV/STDs, HIV testing, Condom use

## Abstract

**Background:**

Behavioral intervention is a key approach to HIV prevention among men who have sex with men (MSM). Widespread use of mobile phones provide us with novel opportunities to decrease HIV infection and transmission of MSM. The objective of the study was to design and develop a mobile phone application (app) aims to conduct behavioral intervention to MSM and to evaluate the efficacy of the app-based intervention compared to usual care, to analyze cost-effectiveness and mechanism of the intervention.

**Methods:**

This study involves 2 phases, phase 1 use qualitative method and phase 2 is a randomized controlled trial lasting for 18 months, they will be conducted in Chagnsha, Hunan Province, China. Phase 1 is to design and develop the app, procedures including retrieval of domestic apps related to prevention and treatment about HIV and sexually transmitted diseases (HIV/STDs), personal interviews with MSM about preferences and functional needs of the HIV prevention app, multidisciplinary experts focused group discussions of the app, software engineers’ development and users test of the app will be performed. In phase 2, we will recruit 800 MSM by cooperating with the local center of disease control and prevention and nongovernmental organizations, and divide them into intervention and control group evenly. Intervention group participants will receive app-based HIV prevention. Control group participants will be provided with usual care including HIV/STDs knowledge brochure and free voluntary counseling services. Data will be collected at baseline, 6, 12 and 18 months since subject’s participation. Effectiveness of the intervention includes HIV/STDs infection rates, adherence to regularly HIV testing, sexual risk behavior, consistent condom use and relative risk of HIV infection. Cost-effectiveness will be analyzed by decision-analytic modeling, and mechanism analysis of this app-based intervention will be performed by path analysis.

**Discussion:**

This will be the first study of its kind in China to develop an app and implement app-based HIV prevention intervention among MSM. It is of great potential to determine whether app-based intervention is a cost-effective way to decrease HIV infection among MSM and explore intervention mechanism with an accurate method.

**Trial registration:**

Chinese Clinical Trial Register (ChiCTR-IOR-15006724). Registered 10 July 2015.

## Background

Men who have sex with men (MSM) have been identified as high risk population of HIV infection and transmission since the world’s first AIDS case found in the United States at 1981 [1], and studies about HIV prevention among MSM had not been paid enough attention by Chinese researchers until the first case of AIDS patient in China found at 1985 [2]. HIV prevalence among MSM population has been continuously increasing at a rapid speed [3, 4]. In China, the proportion of HIV infection among MSM population jumped from 1.4% in 2001 to 6.3% in 2011 [5]. In 2007, MSM accounted for 12.2% among all of the yearly found new HIV infection cases, but this percentage soared to 29.4% in 2011 [6]. And in Changsha, Hunan Province of China, 46% of the yearly found new HIV cases are MSM (Changsha CDC). MSM have become the key population of HIV prevention interventions.

Despite scientists’ ongoing efforts to explore biomedical techniques of HIV prevention such as vaccine development, circumcision, antiviral drug use and so forth [7–9], behavioral interventions are still regarded as key approaches to HIV prevention [10, 11]. These involve regularly HIV testing, free condom providing, health education (e.g. peer education), individual behavioral and mental health counseling [12–17], etc. They have been proved to be effective of improving MSM’s health behaviors, finding newly infected individuals timely and referring them to rapid care and treatment services. Lots of existed studies involved an intervention package which consists of more than one way of behavioral intervention [18, 19]. However, since most of the interventions were performed by face-to-face patterns, there were three limitations which may constrain their effects. (1) High financial cost, labor consumption, and time consuming, (2) narrow coverage of MSM group, and (3) unsatisfying intervention adherence [20]. So it is imperative to explore more effective, extensively accessible, economic and comprehensive HIV prevention tools and strategies [21].

The contemporary world has been greatly changed by the Internet. And the Internet supported interventions have been regarded as “*disruptive innovations*” and become new weapons by researchers and health providers when it comes to HIV prevention [22]. The internet-based interventions have 3 irreplaceable advantages over face-to-face delivered interventions, (1) these interventions can be broadly diffused and maintained at lower financial and personnel cost, (2) the Internet’s characteristics like anonymity and information confidentiality can improve acceptability of the interventions among MSM, (3) the interactive functionality of the Internet gains popularity among different populations, especially among those hard-to-reach groups [23, 24].

Online surveys can provided us with a large amount of information about MSM’s sexual behaviors and mental health in a short period of time, giving auto reminders of HIV testing to clients, conducting motivational interviews about behavior changing and health skills promoting, and performing health education by using websites [25–28]. Levine and colleagues developed an online partner notification system *inSPOT* to inform sex partners of the HIV-positive person to take HIV test [29]. In China, gay websites often be used as tools for health behavior collecting, education delivering, and as a recruiting method for intervention outreach to offline sites [30–32]. Guangzhou CDC of China cooperated with a gay website named Guangtong website to develop a web platform named *Easy Tell* to do anonymous partner notification [33]. Effects of these Internet-based HIV prevention interventions were proved to be comparable to those face-to-face interventions, and they were more accessible and acceptable.

Mobile network is the combination of mobile communication devices and the Internet, mobile phone application(app) is the typical type and current trend of mobile network [34]. Six-hundred fifty-six million Chinese are currently using mobile phones to link to the Internet [35], and it is estimated that 6.72 to 16.8 million of them are MSM. Comparing with computer network, mobile network has more strength, especially with the users online almost 24/7, we can make the intervention to penetrate into the users daily lives. And because of the personal privacy of mobile phone, the intervention can be more targeted and individualized. Besides, personal behavioral data can be collected easily by mobile network due to its portability and access to sensing devices, the big data collected can be analyzed to explore the mechanism of the intervention, which previous studies cannot hold a candle to.

It was consumed that app-based interventions have great potential to become a vital window for health maintenance and promotion of MSM [36]. Several qualitative studies conducted by MSM focus group discussions demonstrated preferences and functionality of HIV prevention app. The app should include educational content like information about HIV testing and prophylaxis distribution institutions and groups, HIV/STDs diagnose and treatment information, drug and alcohol abuse risk and safer sex skills. Interactive engagement such as connection with gay-friendly health providers, other HIV-positive gay men, and support peers/groups. Usability of the app is the most important feature because the app would not be of value if MSM do not use it, which means MSM feel useful, safe and trustworthy about the app’s language and confidentiality [24, 37, 38]. Our previous studies found that mobile phones were widely used among MSM, it was popular for MSM using the gay tailored dating apps such as Grindr, Jack’D, and domestic apps like Blued, ZANK for speed dating, casual sex, or even group sex. Lots of Chinese MSM expressed urgent need of humanistic care, professional mental and behavioral health services, and welcome interventions using the app, Wechat and other mobile network media with open arms [39, 40]. However, there is no MSM tailored, app-based HIV prevention emerged so far.

The objectives of the study are to (1) design and develop an easy to use, user-friendly, non-stigmatized and free app, and deliver app-based HIV prevention intervention to MSM, and (2) to evaluate the effectiveness of the app at decreasing rate of HIV infection, promoting HIV testing behavior and consistent condom use among MSM, and cost-effectiveness analysis of the intervention. App-based intervention mechanism will also be explored.

## Methods

### Study design

This study comprises 2 phases. In phase 1, qualitative method is employed for designing and developing the app. Functional modules of the app are designed on the basis of (1) a retrieval of currently exist HIV/STDs prevention and treatment related apps, (2) personal interviews with MSM about preferences and function needs of the app, and (3) periodical meetings of a multidisciplinary team. Fifty MSM will be invited to test the app after completion of app development and modifications will be made according to their use experience and advice. Phase 2 is a randomized controlled trial lasting for 18 months, to evaluate effectiveness of the app-based intervention and to explore intervention mechanism. Eight-hundred MSM will be recruited and evenly divided into the intervention arm and control. Participants in the intervention arm will receive app-based HIV prevention intervention, and the control arm individuals will receive usual care service which includes free health education materials, voluntary counseling and testing services.

### Setting

Subjects recruitment will be performed in Changsha, provincial capital of Hunan province, China. The city has over 6 million people, including 15 thousand MSM. Implementation of this study will be cooperated with Chagnsha CDC. It is the authoritative HIV testing institution, and has the right to verify one’s HIV-positive status. In addition, the center takes charge of two non-governmental organizations (NGO) which exclusively providing HIV testing and counseling services for MSM. These organizations have built good relationship with MSM.

## Participants

### Inclusion and exclusion criteria

Participant inclusion criteria: (1) male aged 16 years or older, (2) has experience of same-sex anal intercourse during the last 12 months, (3) HIV negative, (4) own at least one smartphone, and (5) willing to report individual HIV/STDs testing results to the researcher. Participant exclusion criteria:(1) has only one sex partner who is also HIV negative, and their relationship has last for more than 2 years, (2) unable to participant the program because of cognitive or psychiatric disorders, or (3) has already attended another intervention program.

### Recruitment

For the participant enrollment, in phase 1 and phase 2, MSM who are potential eligible for the study will be listed, and each individual will be invited to take free HIV testing and participate in the study through phone call performed by the staff of CDC and the two NGOs. An appointment will be made if the person agrees to participate. Following an oral informed consent, clients will be screened for eligibility as subjects of the study by a research member, demographic information and baseline data(details below) will be obtained, and blood samples will be taken for HIV testing. Written informed consent will be obtained from each one who is HIV-seronegtive and willing to participate in the study. Those HIV-seropositive persons will be referred to local CDC for verification and treatment.

### Randomization and blinding

Participants in the randomized controlled trial will be assigned 1:1 to the intervention and control arms under restricted randomized block design (the block length is 4) after informed consent and collection of baseline data. The allocation sequence will be generated and released to the interventionist on a case-by-case basis by another independent department specializes in generating research random sequence. The recruitment staff have no access to the results of randomization prior to recruiting the subject. Participants will be informed of his group by a research assistant by phone. Interventionist, data collection staff, statistician are not the same persons. This is a single-blinded trial. The subjects cannot be blinded due to the nature of the intervention, and interventionist also acknowledge all those contacted are in the intervention arm. But anonymous responses will be entered into the database by a person unconnected with the project. The statistician will be blinded to individual result during the intervention period, and the allocation-to-trial-arm coding will not be revealed until dataset is sealed. The interventionist and supervisors will be blinded to the baseline and follow-up outcomes.

## Procedures

### Phase 1: Development of the app

First, on the basis of our previous studies and references reviewing, preliminary functional modules of the app were presented in Table 1 below. Next step of this study is to search and evaluate currently exist domestic HIV/STDs prevention and treatment related apps on Apple.

**Table 1:** Function modules of HIV prevention app for MSM

Name	Function description
Behavioral assessment	Assessment about user’s unprotected sex behaviors and condom use rate
Behavior change plans	Setting personal goals and plans of health behavior change, and individualized reward/punishment plans
Counseling	Buttons for enter into phone, message, Wechat and email counseling areas
Health education	Knowledge about HIV diagnosis, prevention and treatment, skills of condom using, etc
Personal health records	Uploading HIV/STDs testing results, building personal health records and health promotion plans
Information push	Delivering latest news HIV prevention, treatment and related policies, and reminders of personal behavior, encouragement and advice
Experts encouragements	Videos from professionals and celebrities to encourage user’s adherence to behavior change efforts
Institutions introduction	Providing information about addresses, connection methods, providers and services offered of CDC, hospitals, NGOs nearby
Feedback platform	Building a communication platform where users can give feedback of using the app
Setting	Settings of time period of receiving information, passports of log in the app, latest versions, etc

Store and Android Market since the two markets occupy 90.61% of the current mobile application markets [41]. We use the words “HIV/AIDS/human immunodeficiency virus/STD/ sexually transmitted disease/sexual health/sexual behavior/positive sex/condom” respectively to search for related apps, each eligible app will be downloaded and its features, including names, download numbers, target population, main function modules will be listed and analyzed.

Second, personal face-to-face interviews with MSM will be conducted by using a semi-structured outline, which involves questions about currently confronting behavioral, mental and social problems, promotion and barrier factors of taking HIV test, ever used or currently using MSM tailored apps and their characteristics, preferences of name, logo, and functional needs about a HIV prevention app especially for MSM. Sample size of the interview process will depend on the information saturation rule which means as sample expanding, no new theme be induced from information the participants supplied. A functional framework will be built based on the two processes.

Third, periodical focused multidisciplinary experts group sessions will be held to discuss the framework of the app from the interactivity, feasibility, acceptability and user experience aspects. Finally, functional modules of the app will be determined. The app will be developed by a company specialized in mobile application development. After the app development completed, 50 MSM will be invited to test it, necessary modifications will be made according to their advice.

### Phase 2: Effectiveness evaluation of the app-based intervention

#### Intervention description

Participants in the intervention arm will receive app-based intervention. Each of them will be required to download the app and register as a user consequently, their accounts information, e.g. account name, email address used for registration will be collected. Four disciplines will be taken into account during the intervention period: (1) Interaction: at least once interaction will be required between the user and the app per week (e.g., sexual risk behavior report). (2) Non-stigmatization: a supportive atmosphere including social, behavioral, and psychological support will be built, and all contents will be presented without sense of discrimination. (3) Motivation encouragement: design and development of the app, information offered by the app will be in line with users’ needs, willingness and preferences so as to inspire their using motivation and maintain their adherence of the intervention. And two methods, health behavior goals setting according to the user’s sexual risk behavior, and exchange for free condoms with points earned by using the app regularly will be involved as incentive approaches to increase individual’s motivation of behavior change. (4) User-experience-centric: the app-based intervention will be user-oriented and we lay emphasis on user experience from aspects of interface and function modules as well.

#### Control group

For the control arm participants, each of them will be asked to build connection with the researcher through Wechat, the most widely used instant massaging app in China. They will receive usual care including free brochures of health information and free counseling services whenever they need. All of the participants will be promised of the confidentiality throughout the process.

#### Participant follow-up

All of the subjects in control and intervention arms will attend four visits, one at baseline for screening and enrollment, three at 6-, 12-, 18-month respectively to assess their sexual and other health behaviors (detailed below). Any subject gets HIV infected during the whole study period will be referred to professionals for timely treatment and withdrawn form the study. Information and psychological counseling services, positive sex tips and necessary links to care resources will be provided by the study staff.

**Fig. 1:**
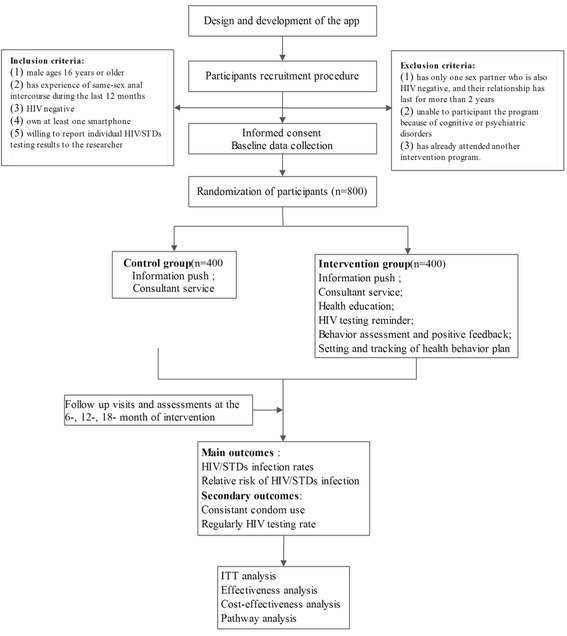
Participant flow chart

Figure 1 is the Participant flow chart of the study.

#### Data collection

Baseline data of all the subjects will be collected at the enrollment procedure. The measures including demographic information (*age, marital status, current occupation, educational level, income and sexual orientation*), sexual behaviors (*number of male and female sex partners, frequency of sex, sex role, commercial sex behaviors, sex with fixed and casual sexual partners*), and health-related behaviors (*consistent condom use with multi-type of sex partners, regular HIV testing, drug use experience and sexual transmitted diseases infection history*).

For the rest three times of data collection, sexual behaviors, health-related behaviors, and app user experience (*user experience, feedback and suggestions*) during the last 6 months will be involved in an electronic questionnaire. One version will be made into the app and be delivered every 6 months to intervention arm participants. Another without app user experience items will be delivered by Wechat to the ones in control arm.

Data of cost of the intervention and participants’ app use behaviors will also be collected. Direct cost involves cost of development, operation and maintenance of this app and worker cost, indirect cost consists of participants’ expenses due to their joining in the study such as time spending, cell phone internet data and traffic cost. App use behaviors will be collected through the management system which involves participants’ use frequency and time durance, information scanned, interaction times and frequency.

We use the electronic questionnaire to make sure that all of the items will be presented in a logical order and necessary answers missing will not be allowed. The participant will be offered a non-cash financial compensation as phone bills worth 20 RMB for compensation every time he complete the data collection.

#### Outcome measures

Outcomes will be compared between intervention and control arms, and between different time points of each arm. The primary outcomes include HIV/STDs infection rates, relative risk of HIV/STDs infection, secondary outcomes include condom use rates, adherence to regularly HIV testing. The incremental cost is defined as per HIV infection case decreased according to the app-based intervention, cost of every 1% decrease of HIV/STDs infection, or every 10% increase of consistent condom use and adherence to regularly HIV testing. Another outcome is the app-based intervention mechanism (detailed below).

#### Sample size and calculation

The intervention arm and control arm are of equal size and two-tailed hypothesis testing is assumed. Sample size is calculated by using the specific sample size function module PASS on the statistical analysis software NCSS [42]. By setting the α = 0.05, 1-β = 0.9, effect size as 0.25 [43], the sample size of each group is 336. With an estimated 20% loss to follow-up, 400 subjects are necessary for each group.

### Statistical methods and analysis

#### Analysis of equivalence of two arms at baseline

The equivalence will be analyzed by using *Mann-Whitley U* test, *Chi-square* test, *t* test or *ANOVA*.

#### Analysis of intervention outcomes

For intervention arm and control arm comparison, indicators of rates of HIV/STDs infection, consistent condom use, adherence to regularly HIV testing and sexual risk behaviors at baseline and at 6-, 12- and 18-month will be compared by using Chi-square test. And a COX risk model will be used for predicting HIV/STDs infection risks.

For the cost-effectiveness analysis, the total cost consists of direct and indirect cost mentioned above. Effectiveness covers the increased rates of consistent condom use and regularly HIV testing, the decreased rate of HIV/STDs infection, relative risk of HIV/STDs infection. A decision analytic model will be used to evaluate economic evaluation of the intervention. Using the estimated effectiveness and incremental cost of per HIV infection case decreased, every 1% decrease of HIV/STDs infection, every 10% increase of consistent condom use or adherence to regularly HIV testing, incremental cost-effectiveness ratio will be calculated.

#### Intention-to-treat (ITT)

Outcome will be analyzed under the basis of ITT. First, the principles of ITT will be applied to test the effects of attrition, which means that all participants who have been randomized will be included in the analysis. Missing endpoints will be imputed by using the Expectation-Maximization (EM) algorithm. To gauge the robustness of the outcomes, this analysis will be repeated while using Multiple-Imputation approach. Second, propensity Score analysis will also be used. By using logistic model (1 for adherence and 0 for attrition) to predict the probability of be in adherence. And then use the predicted variable to replace the attrition dummy variable.

#### Analysis of intervention mechanism

Correlation analyses between each aspect of participants’ app use behaviors (e.g. app use frequency and time durance, information scanned, interaction frequency) and effectiveness of the intervention will be performed. A causal path model will be built according to correlation analyses results and related theories. And a path analysis will be subsequently conducted so as to calculate the residual path coefficient and determination coefficient, define causal relationships between different variables, and thus explore intervention mechanism of the app-based intervention.

## Discussion

In line with goals of implementation science, the study is going to develop a mobile phone app which comprises of behavioral, psychological and social services and for MSM, and implement a randomized controlled trial of HIV prevention intervention by using this app among MSM and evaluate effectiveness of the app-based intervention. The goals of the intervention are to provide MSM with health information, encourage their motivation of behavior change and promote their health skills.

It has been shown that apps are beneficial to self-management of people with chronic conditions, physical activity promotion among different age groups, and other health related behaviors [44–46]. There were systematic reviews pointed out that apps have great potential as tools for HIV prevention among MSM, and there were also studies on MSM’s acceptability and preferences of app-based. However, there is no randomized controlled trial targeting at MSM group with an app. This is the first study of its kind.

There are several strengths of the study. Mobile network is the combination of the Internet and mobile devices, and has the advantage of higher portability. The “always online” feature made it become a part of one’s daily life. Mobile phone can protect personal privacy and realizes point-to-point individualized intervention as well, which provide us with a better and accurate way to illustrate intervention mechanism. The long-term follow-up makes it possible to observe intervention effects with more persuasive results. And the cost-effectiveness analysis will help policy makers perform informed and practical decision-making.

As for the limitations of the study design, due to the open design we cannot mask the intervention, participants will be aware that they are in a HIV prevention intervention, which may introduce bias. Randomization allocation of participants into different groups can help achieve balance in sociodemographic characteristics. And researcher in charge of statistical analysis will be blinded to group allocation.
